# In Situ Sensor for the Detection of Oil Spill in Seawater Using Microwave Techniques

**DOI:** 10.3390/mi13040536

**Published:** 2022-03-29

**Authors:** Aliyu Dala, Tughrul Arslan

**Affiliations:** Integrated Micro & Nano Systems, University of Edinburgh, Edinburgh EH8 9AB, UK; t.arslan@ed.ac.uk

**Keywords:** microwave, underwater antenna, PDMS, crude oil, sweater, Faraday cage, radio frequency, reflection coefficient, transmission coefficient

## Abstract

Nearly 30% of oil drilled globally is done offshore. Oil spillage offshore has far-reaching consequences on the environment, aquatic lives, and livelihoods as it was evident in the Deepwater Horizon and Bonga oil spills. A novel microwave in situ oil spill sensor was developed in this work. The device is comprised of two polydimethylsiloxane (PDMS)-encapsulated ultra-wideband underwater microwave trefoil antennas enclosed in a Faraday cage with one serving as the receiving antenna and the other as the transmitting antenna. Heavy aromatic-naphthenic Azeri crude oil was used as an inclusion in the seawater host medium. Substantial changes in the measured reflection (S11) and transmission (S21) coefficients were observed as the medium was adulterated with crude oil starting from 200 MHz to around 2500 MHz. The changes in the dielectric properties of the media resulted in changes in both the S11 and S21 signifying and detecting an occurrence of the oil spillage. Thus, by employing radio frequencies, oil spillage was detected using the in situ monitoring device in seawater.

## 1. Introduction

Humans have been using crude oil in one form or another for thousands of years. From the ancient Egyptians that used it to mummify their dead to the Babylonians that waterproofed their boats with it [1]. However, the real potential of crude oil was unleashed in the mid-19th century. The modern history of the world is entwined with that of oil. The Industrial revolution, the invention of the automobile, and the world wars were all powered by oil. It has been able to uplift countries from squalor to prosperity in an instance. We live in an oil world; we are surrounded by it. Oil is presently used to power our industries, automobiles, aviation, homes, and offices. The derivatives of crude oil have permeated every sphere of our lives. From some of the plastics we use to some synthetic rubber, cosmetics, chemicals, and lubricants, the importance of crude oil cannot be overemphasised [2].

Oil has an edge over other energy sources in that it is concentrated and could be easily transported over long distances. Sometimes, during these transportations, accidents do occur. For example, the largest accidental oil spill in US history, BP’s Deepwater Horizon oil spill [3]. Oil spilled into the Gulf of Mexico from April to September 2010. This resulted in the loss of lives of 11 workers with 134 million gallons of oil spilled resulting in about 2100 km of the U.S. Gulf Coast covered in oil. British Petroleum (BP) was forced to pay $65 billion as settlement. The incident was as a result of the failure of the blowout preventer (BOP) [4], which was connected to the riser for oil drilling from the well. The BOP was supposed to seal the oil well to prevent the spillage when the rig exploded but it failed to do so.

Around a year later, a similar incident occurred off the coast in the Gulf of Guinea. The Bonga oil spill is one of the largest in Africa involving Shell Nigeria Exploration and Production Company’s (SNEPCO) in Bayelsa, Nigeria [5]. The pipeline connecting the well to the float production storage and offloading (FPSO) ruptured and oil was released into the sea. Around 40,000 barrels gushed into the Atlantic covering an area of about 950 square km [6]. Shell was fined $3.6 billion for the damage and the loss of livelihoods for the communities that depended on the area for their sustenance and the death of numerous marine lives.

A robust monitoring system for oil spill detection would have been very useful in the early detection of the oil spill in both cases highlighted above and for most of the countless incidences of oil spills that have happened. We shall now discuss the different solutions that have been employed for remote monitoring of oil spills.

Several techniques have been used over the years for the detection of oil spillage. These methods could be broadly classified into two: active and passive remote sensing, depending on whether the sensors emit signals (active) or if it relies on signals generated by the environment to sense (passive).

The obvious limitation of the passive sensor is that it needs the perpetual presence of the source of illumination or signal to be able to remotely sense. Thus, for example, at night or in the absence of the sun, most passive sensors fail to detect except in cases of a thermal infrared.

The two most popular forms of active remote sensing are RADAR, which stands for radio detection and ranging, and LIDAR, light detection and ranging. RADAR sends out microwave or radio signals at a target and measure the reflected signals for detection. In the case of LIDAR, the emitter transmits light waves and receives the reflected signal using a collector to detect any changes.

For marine or ocean remote sensing, there are distinctly two modes of oil spill detection: water surface and underwater remote sensing [7]. The first and most frequently deployed is the water surface remote sensing. Numerous types of passive and active remote sensing methods deploy techniques such as visible spectrum, infrared, near infrared, ultraviolet, and microwave. The latter, microwave (RADAR), is the most popular method. There are three main configurations of radar which are side-looking airborne radar (SLAR), synthetic aperture radar (SAR), and a third less used type, ship-borne radar. Each has its advantages and demerits. SLAR is cheaper and mostly used in airborne systems such as an aircraft. The ship-borne radar is used on ships offering a range of 3 to 80 km depending on the antenna height.

SAR has wider coverage and offers better resolution. It is often used on satellites for remote sensing. SAR is an active remote sensing technique that involves mounting radar on a straight-line moving platform, which could be an airplane or a space-borne system such as satellite. This radar is directed at an area of interest to produce fine-resolution images in three dimensions or two dimensions. Similar to any imaging radar, an electromagnetic signal travelling at the speed of light targets a surface. The signals are reflected from the surface as a backscatter which is recorded as well as the time delay. The SAR image is developed using the strength of the backscatter and its time delay [8]. This technique has been extensively used in the detection of oil spill in marine and coastal areas. Some of the popular operational satellite SAR include RADARSAT owned by the Canadian Space Agency, Sentinel-1 a collaboration between the European Commission (EC) and European Space Agency (ESA), Kompsat-5 managed by the Korean Aerospace Research Institute (KARI), TerraSAR-X controlled by German Aerospace Center, and TecSAR developed by Israel Aerospace Industries Ltd.

In [9], SAR was used for the detection of marine oil spill over the Indian Ocean using four oil spill events as case studies. An improved methodology using S-1 SAR satellite data at speeds between 3 and 9 m/s for all events were utilised. Varying atmospheric conditions and influence of wind currents for oil spill spreads and degradation were investigated. The oil spills’ trajectory production was modelled using the General National Oceanic and Atmospheric Administration (NOAA) Operational Modelling Environment (GNOME) model. The oil spill weathering processes were modelled using automated data inquiry for oil spills (ADIOS). A maximum oil spill movement of 33 km from the source of the spill was observed whereas the evaporation of the crude oil was observed to be high. The study concluded stating the cost effectiveness of the SAR-based oil spill detection technique.

SAR and underwater gliders were used in combination to detect oil seeps in the lower Congo basin [10]. One SAR image of the site was captured at least after every 12 h for a period of 21 days. Two concomitant underwater gliders were fitted with fluorescence sensors and the results obtained from them were compared with those from the SAR images. A total of 80 recurring oil-seeping sites were identified using SAR. Six out of those sites were investigated using the underwater gliders. Consequently, vertical pipes of hydrocarbon fluids detected by the gliders corresponded to the images obtained from the SAR.

In [11], the weakness of the satellite SAR with respect to its inflexibility based on time and location were highlighted. It is one of the most used methods of oil spill detection; however, it suffers from long latency based on when the oil spill occurs and the time when a satellite can send an image on the site and its inability to continually track the spillage. Thus, they proposed the use of an airborne L-band, low noise, high resolution uninhabited aerial vehicle SAR (UAVASAR) for oil spill response. It was able to get several more images in an hour compared to the satellite SAR. Notwithstanding, the limitations of the UAVASAR were identified in the study. Being a science instrument and not meant as an urgent response platform, there is the need for the oil spill response community to develop and deploy airborne SARs in the form of a large aircraft capable of long-range communications or smaller aircrafts for targeted area of coverage. This would make the airborne SAR more expensive than the satellite SAR because the response community does not invest in the satellite SAR expeditions.

To mitigate against the weakness of the satellite SAR, a proof-of-concept model for the use of multiple sensors, including satellite SAR, in situ measurements, and multispectral imaging for the detection of oil emulsions, was put forward in [12]. Previous traditional techniques of using SAR were in the detection of presence or absence of surface oil rather than in the determination if the oil type was emulsified or not. Their research work’s contribution was the ability to discriminate oil emulsions within an oil slick. For the in situ measurements, three different techniques were explored for measuring the oil thickness. The first was the use of absorbent pads, which were suitable for thin-layered oils up to a few hundred μm thick. The second involved the use of dip plates, which depended on the level of emulsification for its performance. Lastly, an automated water mapping oil thickness sampler (WM-OTS) capable of measuring oil thickness from 5 μm to several cm was used. The WM-OTS was selected due to its broad range of operation and consistency.

The challenges and pitfalls of oil spill detection by imaging using imaging radar was reviewed in [13] where the difficulty of discriminating between radar signatures of oil films and biogenic slicks were highlighted. These often led to misleading results obtained for the oil spill detection. With satellite SAR, the preponderance of false positives for oil spills that are misinterpreted and false negative where there is no detection, when, however, an oil spill has occurred inspired [14] to adopt the use of an in situ autonomous system for the detection of oil spill. The proposed system termed ARIEL consists of a drone and an unmanned surface vehicle (USV). Both systems work in a collaborative fashion with oil detection sensors installed in each of them. The drone, the first layer, was installed with a visible and a thermal camera package. It was used to eliminate false negatives. The second layer, the USV, was fitted with a *fluorosensor*. It validated all cases reported by the drone and also detected unnoticed cases. The Atmospheric Remote-sensing Infrared Exoplanet Large-survey (ARIEL) system aimed to reduce the cost associated with deploying manpower in cases of false positive and the corresponding human risks. However, the configuration of this system makes it expensive, and the cost of maintaining a system such as this could become exceedingly high.

In [15], the PRogetto pilota Inquinamento Marino da Idrocarburi project (PRIMI) was combined with a forecasting module and an observation module responsible for the oil spill detection based on SAR and LIDAR, to detect oil spill and forecast the oil spill displacement after the detection. The forecasting module was based on Lagrangian numerical circulation models. The in situ models were based on simulation. Several oil spills were detected using the observation modules. These spills were verified in situ using the forecasting modules. In this study, a case for further work to combine the satellite models and realistic in situ data to refine the PRIMI data was made.

The underwater remote oil spill sensing also involves the use of both passive and active sensors [7]. Some of these techniques are used in the detection of oil in the water column or at the bottom of the sea. One of such techniques employs the use of ultrasonic to detect oil spill based on the difference in the acoustic profile of water and oil at the bottom of the sea. Laser fluorosensors have been used for the detection of oil spill up to a distance of 2 m in the water column. It does this by detecting the aromatic compounds found in oil. Chemical analysis, comprising of spectrometry or fluorometry, have been used to also detect oil in water. The use of camera for the detection of oil has also been employed.

The detection of oil spill with respect to thermal IR depends on the temperature difference between the emulsified oil and the surrounding water. During daytime, the sun heats up the water surface; however, the high viscosity of the emulsified oil means that internal convection is restricted. Thus, the emulsified oil layer does not lose heat to the underlying water surface and, therefore, is warmer. These changes, however, disappear at night except in the few circumstances that the air temperature is substantially warmer than the water surface temperature. Here in lies the limitations of this technique [16].

A spill oil point-of-testing device (SOPD) was developed in [17] for on-site fluorescence monitoring of oil concentrations. SOPD adopts a multi-mega pixel approach, which can detect oil spill even in the presence of environmental noise caused by dust and other impurities. This has superior performance compared to photodetectors that are commonly used in existing instrumentation that rely on single-pixel detection. It uses light-emitting diodes (LEDs) as the excitation source and a complementary metal-oxide-semiconductor (CMOS) image sensor as a detector.

In this research work, we designed and developed a novel in situ radar-based oil spill detection sensor for underwater application including deployment on an oil riser, which connects a floating production storage and offloading (FPSO) or oil rigs to the oil wells and have been responsible for numerous oil spills including the Deepwater Horizon and Bonga oil spills.

The monitoring device, comprising of a Faraday cage and polydimethylsiloxane (PDMS)-encapsulated microwave antenna, would be capable of detecting oil spill in real-time. To the best of our knowledge, after reviewing the literature for state-of-the-art, we have not come across any in situ remote sensor that employs microwave techniques for the detection of oil spillage in an underwater environment. Our system could be used in conjunction with satellite or airborne SAR for the detection of oil spill, as well as tracking the movement of the oil slick.

## 2. Materials and Methods

### 2.1. Antenna Design

Due to the varying and contrasting values of the dielectric properties of freshwater, seawater, oil, gas, and brine, the candidate antenna should be capable of operating over these different materials As an example of the divergent relative permittivity of some of the materials, that of water is 70–j10 while that for oil is 2.2–j0.1 [18].

A wideband antenna characteristic would be essential for optimal performance over these ranges of materials, especially in order to be able to adapt an imaging or detection algorithm for radar-based microwave sensing such as Confocal Delay and Sum (CDAS) [19], for a subsequent imaging and determination of oil slick thickness. Another essential characteristic of the antenna would be compactness. This quality is readily offered by patch antennas that exhibit good performances at relatively smaller dimensions. They are also lightweight, easy to fabricate, cheap, and could also be adapted to be used in any shape. These attributes make this antenna a very suitable option. Based on these requirements stated previously, we proceeded to use an ultra-wideband PDMS-encapsulated trefoil antenna. The trefoil design enabled the antenna to have ultra-wideband characteristics both in free space and when encapsulated and submerged underwater.

**Figure 1:**
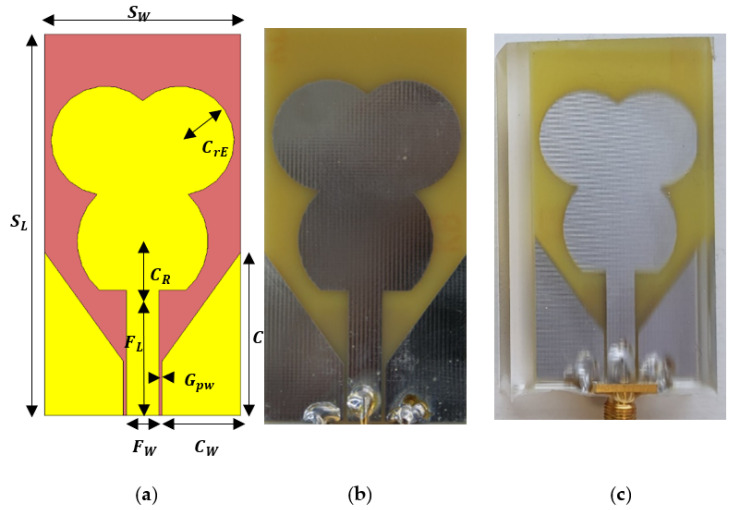
Trefoil antenna: (**a**) CST simulation model, (**b**) fabricated, (**c**) encapsulated in PDMS.

The computer simulation technology (CST) model developed in the electromagnetic field simulation software and the fabricated trefoil antenna used in this experiment can be seen in Figure 1a,b respectively. The antenna was fabricated on FR-4 substrate with relative permittivity of 4.3. The substrate thickness was 1.6 mm, the dimension was 70.5 mm by 36.5 mm, the copper thickness was 1 oz with no soldermask. The PDMS-encapsulated trefoil antenna can be seen in Figure 1c. The dimensions of the antenna can be seen in Table 1.

### 2.2. Faraday Cage Setup

**Figure 2:**
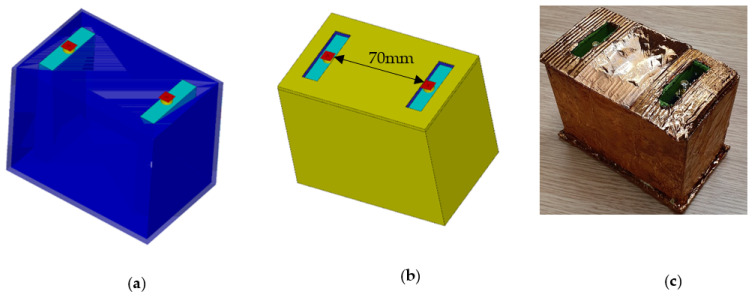
Oil spill sensing region at 70 mm antenna spacing: (**a**) acrylic, (**b**) Faraday cage in CST, (**c**) fabricated Faraday cage.

Two PDMS-encapsulated antennas were placed inside an acrylic container of 2 mm thickness in the CST environment. The container was filled with water at 20 °C. The distance between the antennas were varied and the reflection coefficients and transmission coefficients were recorded. The antenna distances of interests were 15 mm, 20 mm, 40 mm, 60 mm, 70 mm, and 80 mm. A Faraday cage used to eliminate interference from external electromagnetic waves was developed in CST by covering the acrylic container with copper. The simulated model of the acrylic container and the simulated and fabricated Faraday cages could be seen in Figure 2a–c, respectively, for the 70 mm antenna spacing all filled with seawater.

### 2.3. Oil Spill Simulation

To have a proof of concept for the oil spill detection, the Faraday cage with 15 mm antenna spacing was used as the experimental setup. It was filled with a medium with variable relative permittivity. The relative permittivity property of the medium was varied to mimic that of the oil spill. Dielectric constant values between that of an unadulterated freshwater at 78 and seawater at 74 to that of an absolutely adulterated crude oil at 2.33 were chosen. The values of the relative permittivity between these extremes were 68, 58, 48, 38, 28, 18, and 8. The result and implication shall be discussed in Section 3.2.

### 2.4. Oil Spill Experiments

With the seawater as the medium and the heavy aromatic-naphthenic Azeri crude oil (HANACO) as the inclusion, two-phase Tinga-Voss-Blossey (2-TVB) mixture model suitable for materials that have multiple confocal ellipsoidal structures that mix poorly with each other, could be used to estimate the dielectric properties of the resulting mixtures [20]. The quasi-two-phase model of the complex relative permittivity of the mixtures has been adopted for seawater given by Equation (1).

(1) $$\varepsilon_{mixture}=\varepsilon_{medium}+\frac{3v_{oil}\varepsilon_{medium}\left(\varepsilon_{oil}-\varepsilon_{medium}\right)}{2\varepsilon_{medium}+\varepsilon_{oil}-v_{oil}\left(\varepsilon_{oil}-\varepsilon_{medium}\right)}$$

where ε is the complex permittivity, v is the volume fraction, $\varepsilon_{medium}$ is the complex permittivity of the seawater host medium, and $\varepsilon_{oil}$ is the complex permittivity of the inclusion, crude oil.

**Figure 3:**
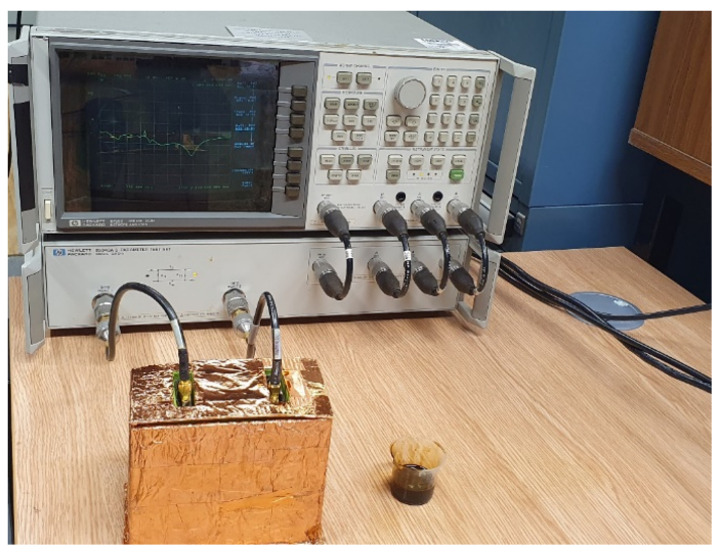
Experimental setup for the oil spill test for crude oil.

The oil spill experiments were performed using seawater medium. The oil spill experimental setup can be seen in Figure 3. A two full port calibration was done on the Hewlett-Packard (HP) 8753 Vector Network Analyser (VNA) using the open, short and load studs. The frequency span was chosen to be 2999.7 MHz with a start frequency of 0.3 MHz and stop frequency of 3000 MHz. The number of points were selected to be 1602 to get very accurate plots. The power was set at −10 dBm. After the calibration, the two ports of the 8753 VNA were connected to the two PDMS-encapsulated antennas. The transmitting antenna was connected to port 1 while the receiving antenna was connected to port 2.

The two antennas were placed in the Faraday cage. We used heavy aromatic-naphthenic Azeri crude oil (HANACO) for the experiment. 650 mL volume of seawater was then poured into the Faraday cage. The S parameters for the unadulterated seawater was obtained using KE5FX VNA Utility software on the PC that was connected to the 8753 VNA via the general purpose interface bus (GPIB). Subsequently, a volume of 10 mL of the HANACO was added to the seawater medium from the top of the Faraday cage continuously until a total of 100 mL of crude oil had been added. The mixture was disturbed to mimic the real-world scenario. The S Parameters of the new medium at each 10 mL crude oil addition was captured. When finally immersed in water, the Faraday cage ensures that the fidelity of the sensor is maintained by excluding external electromagnetic interferences. The result shall be discussed in Section 3.3.

## 3. Results and Discussions

For simplicity, we shall be looking at only the reflection coefficients (S11) of the transmitting antenna and the forward transmission coefficients (S21) of the transmitting antenna to the receiving antenna. This is because the performances of both antennas were similar.

### 3.1. Faraday Cage Setups

**Figure 4:**
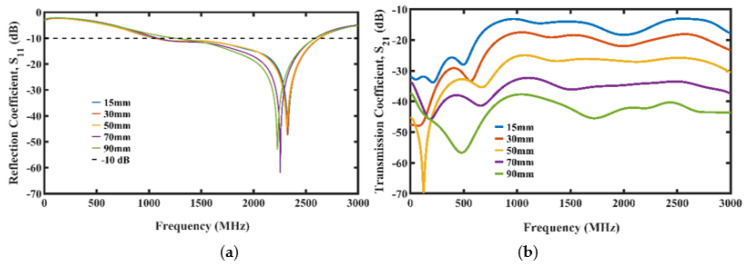
Simulation plot showing different antenna spacing. (**a**) Reflection coefficient. (**b**) Transmission coefficient.

The simulation results for the reflection and transmission coefficients of the PDMS-encapsulated trefoil antennas placed in the Faraday cage filled with seawater with the distance between the antennas varied can be seen in Figure 4a,b, respectively.

Based on the results from Figure 4, we decided to adopt the antenna distance of 15 mm in the Faraday cage for the oil spill simulation and experiments due to its highest S21 value and UWB −10 dB bandwidth from the S11.

### 3.2. Oil Spill Simulation

In this section, the results obtained from the simulation from oil spill in seawater at antenna spacing of 15 mm shall be discussed.

#### 3.2.1. Reflection Coefficient

**Figure 5:**
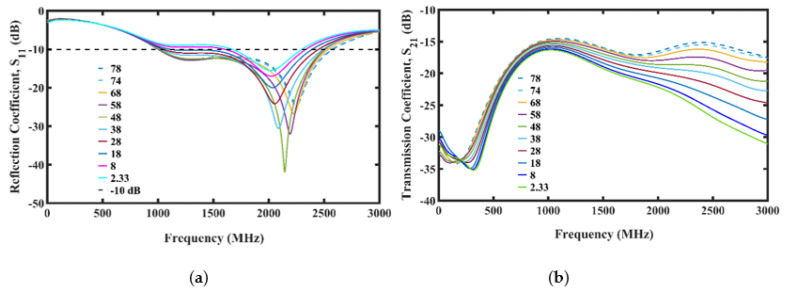
Simulation plot of oil spill. (**a**) Reflection Coefficient. (**b**) Transmission Coefficient.

It can be seen from Figure 5a that noticeable divergences in the reflection coefficients of the different oil spill compared with that of pure seawater began at around 807 MHz. These changes in the reflection coefficients and corresponding shifts in frequency, and consequently bandwidth, is due to a change in the dielectric constant of the water transmission medium because of the oil inclusions.

#### 3.2.2. Transmission Coefficient

The transmission coefficients can be seen from Figure 5b. It can be seen that the value of the transmission coefficients reduces with increasing oil spillage and frequency. Based on the simulation results, the transmission coefficients for the antennas changed with varying measure of the oil spillage.

### 3.3. Oil Spill Experiments

The reflection and transmission coefficients obtained for both the simulation and measured results will be discussed in this section. The measured results are represented as bold lines while the simulated results are shown in dashed lines.

#### 3.3.1. Reflection Coefficients for Oil Spill

Changes can be observed in the reflection coefficient of the antenna-sensors. This was due to changes in the dielectric constant of the seawater that resulted from the introduction of HANACO crude oil, which had a substantially lower relative permittivity compared to the former.

**Figure 6:**
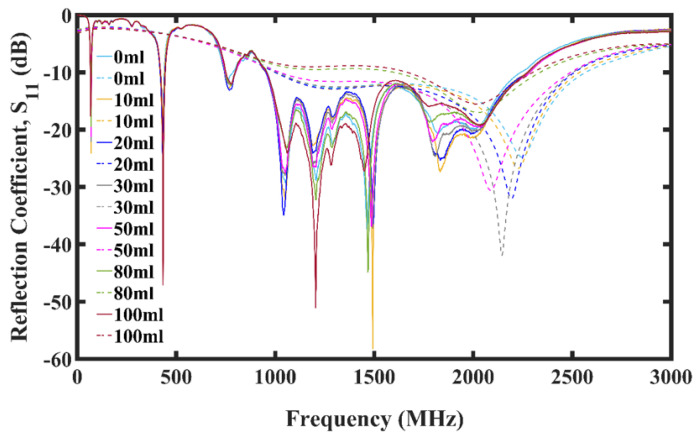
Plot showing reflection coefficient for oil spill using 15 mm antenna-sensors spacing.

For antenna-sensors in the 15 mm setup, the changes in the measured reflection coefficient spanned from 750 MHz to 2500 MHz, as shown in Figure 6. For the simulations, the changes for the antenna-sensors spanned from 800 MHz to 2900 MHz.

These changes in the reflection coefficients and corresponding shifts in frequency, and consequently bandwidth, is due to a change in the dielectric constant of the water transmission medium because of the inclusion of HANACO crude oil.

#### 3.3.2. Transmission Coefficients for Oil Spill

**Figure 7:**
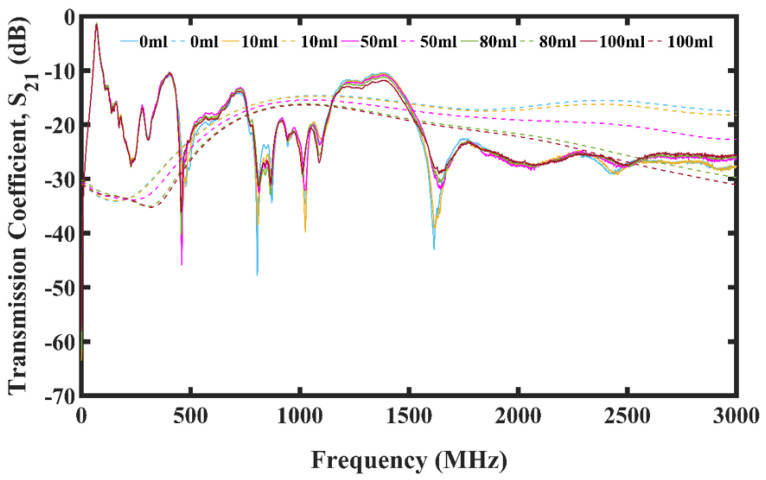
Plot showing transmission coefficient for oil spill using 15 mm antenna-sensors spacing.

The transmission coefficients can be seen in Figure 7 for the 15 mm spaced antenna-sensor Faraday cage setup. The forward transmission coefficient (S21) and the reverse transmission coefficient (S12) were obtained. Because these values were similar for the same setup, only the forward transmission coefficient shall be considered. For clarity, only the S21 for the pure seawater host medium and the mixture with HANACO oil inclusions of 10 mL, 50 mL, 80 mL, and 100 mL are shown.

It can be seen that the value of the transmission coefficients reduces with increasing oil spillage and frequency. Based on the simulation and measured results, marked differences can be seen between the pure seawater and the oil inclusions at 50 mL, 80 mL, and 100 mL. However, the difference between the 10 mL oil inclusion and that of pure seawater was minimal due to the low volume of crude oil inclusion resulting in minimal change in the dielectric property of the mixture compared to the pure seawater.

The difference between the measured and simulation results were because of noises introduced to the measurement from electronics and movement of cables and the use of constant values for the permittivity of crude oil and seawater in the CST simulation software respectively.

## 4. Conclusions

In this research, we have designed and developed a novel in situ oil spill monitoring device capable of the detection of oil spillage in seawater. The device is comprised of two PDMS-encapsulated ultra-wideband underwater microwave trefoil antennas enclosed in a Faraday cage separated by a distance of 15 mm.

Using the reflection and transmission coefficients simulation results, we were able to select antenna-sensors spacing of 15 mm to develop the Faraday cage setup for the oil spill sensing. The use of the Faraday cage was to eliminate electromagnetic interference that may affect the fidelity of the signals. This was done by covering the acrylic container with a copper foil.

We were able to validate the capability of our device for oil spill detection by developing a customised medium in CST with a variable dielectric constant. The relative permittivity was varied between that of fresh water at 78, seawater at 74, and down to that of oil at 2.33. Our sensor was capable of detecting those changes using both the reflection and transmission coefficients. These formed the basis for the experiment.

Due to the high combustibility of crude oil, we decided to use rapeseed oil to provide a proof of concept of the detection capability of our device before proceeding with the crude oil inclusions. After the successful validation with rapeseed oil, we proceeded to use heavy aromatic-naphthenic Azeri crude oil for the oil spill experiment in seawater. The baseline of the pure seawater at 0 mL adulteration was registered and then the crude oil inclusion was continually added to the seawater medium.

The reflection coefficients obtained for the different crude oil inclusions showed variations in the S11 responses in relation to those of the unadulterated seawater medium. It was found that the reflection coefficients were more effective in the determination of the oil spill compared to the transmission coefficient, which had minimal variation of the S21 of the oil inclusions with respect to that of the pure seawater. The developed monitoring device can be deployed in seawater to complement high latency data acquired from airborne or satellite sensing.

The sensor shall be attached to the oil riser or suspended on a buoy in the field. The sensor could be connected to a portable VNA instead of the bulky VNA. With a localised region such as the oil riser or rig, one sensor could be deployed for the detection of the oil spill in that environment.

## Figures and Tables

**Table 1:** Dimensions of Trefoil Antenna.

Antenna Parameters	Symbol	Dimensions (mm)
Substrate Length	$S_{L}$	70
Substrate Width	$S_{W}$	36
Substrate Thickness	$S_{T}$	1.6
Antenna Patch Radius	$C_{R}$	24
Circular Element Radius	$C_{rE}$	10
Feedline Length	$F_{L}$	20
Feedline Width	$F_{W}$	6
CPW Gap	$G_{pw}$	0.59
CPW Length	$C_{L}$	30
CPW Width	$C_{W}$	14.41
Copper Thickness	Tcu	0.035

## Data Availability

Not applicable.
